# Conservation of the TRAPPII-specific subunits of a Ypt/Rab exchanger complex

**DOI:** 10.1186/1471-2148-7-12

**Published:** 2007-02-02

**Authors:** Randal Cox, Shu Hui Chen, Eunice Yoo, Nava Segev

**Affiliations:** 1 Department of Biological Sciences, Laboratory for Molecular Biology, University of Illinois at Chicago, Chicago, IL 60607, USA

## Abstract

**Background:**

Ypt/Rab GTPases and their GEF activators regulate intra-cellular trafficking in all eukaryotic cells. In *S. cerivisiae*, the modular TRAPP complex acts as a GEF for the Golgi gatekeepers: Ypt1 and the functional pair Ypt31/32. While TRAPPI, which acts in early Golgi, is conserved from fungi to animals, not much is known about TRAPPII, which acts in late Golgi and consists of TRAPPI plus three additional subunits.

**Results:**

Here, we show a phylogenetic analysis of the three TRAPPII-specific subunits. One copy of each of the two essential subunits, Trs120 and Trs130, is present in almost every fully sequenced eukaryotic genome. Moreover, the primary, as well as the predicted secondary, structure of the Trs120- and Trs130-related sequences are conserved from fungi to animals. The mammalian orthologs of Trs120 and Trs130, NIBP and TMEM1, respectively, are candidates for human disorders. Currently, NIBP is implicated in signaling, and TMEM1 is suggested to have trans-membrane domains (TMDs) and to function as a membrane channel. However, we show here that the yeast Trs130 does not function as a trans-membrane protein, and the human TMEM1 does not contain putative TMDs. The non-essential subunit, Trs65, is conserved only among many fungi and some unicellular eukaryotes. Multiple alignment analysis of each TRAPPII-specific subunit revealed conserved domains that include highly conserved amino acids.

**Conclusion:**

We suggest that the function of both NIBP and TMEM1 in the regulation of intra-cellular trafficking is conserved from yeast to man. The conserved domains and amino acids discovered here can be used for functional analysis that should help to resolve the differences in the assigned functions of these proteins in fungi and animals.

## Background

In all eukaryotic cells, intra-cellular trafficking connects the cell with its environment by the orderly transport of membranes and proteins via the exocytic and endocytic pathways. In the exocytic pathway, proteins destined to be secreted or presented on the plasma membrane (PM) are transported from the endoplasmic reticulum (ER), through the Golgi apparatus, to the PM. In the endocytic pathway, proteins from the environment or the PM are shuttled via a set of endosomes to lysosomes. The machinery and the mechanisms of intra-cellular trafficking are highly conserved among all eukaryotes (orthologs), and some protein components are also conserved between the various steps of the pathways (paralogs) [1].

Ypt/Rab GTPases are key regulators of this protein trafficking. They are conserved both within a single genome between transport steps and across large phylogenetic distances [2-4]. *Saccharomycescerivisiae *cells contain 11 Ypts, whereas human cells have ~70 Rabs [5-7]. In *S. cerivisiae*, Ypt1 and the functional pair Ypt31/32 regulate entry into and exit from the Golgi, respectively [8,9]. The mammalian Rab1A and Rab1B share ~70% identity with *S. cerivisiae *Ypt1, and Rab1A can replace Ypt1 in yeast knockout cells [10]. The mammalian Rab11A, Rab11B and Rab25 share ~60% identity with *S. cerivisiae *Ypt31/32 and regulate the same transport steps: exit from the Golgi and endosome-to-Golgi transport [11,12].

Ypt/Rabs are activated by specific nucleotide exchangers, called guanine nucleotide exchange factors (GEFs). GEFs for different Ypt/Rab paralogs do not share sequence similarity and are therefore harder to identify. In *S. cerivisiae*, the multi-subunit complex TRAPP was identified as the GEF for both Ypt1 [13,14], and Ypt31/32 [14]. TRAPP is a modular complex that exists in two forms: TRAPPI and TRAPPII [15]. We recently showed that TRAPPI acts as a Ypt1 GEF, whereas TRAPPII functions as a Ypt31/32 GEF [16].

The *S. cerivisiae *TRAPPI was shown to function in ER-to-Golgi transport. It contains seven subunits that co-precipitate as a ~300 kDa complex from yeast cell lysates [15]. There is considerable evidence for the structural and functional conservation of the TRAPPI complex (excluding the Trs85 subunit) from fungi to animals [17] (Table 1).

**Table 1:** TRAPP subunit conservation:

***S. cerivisiae *Subunit** **(#amino acids)**^1^	**Human Subunit** **(# amino acids)**^1^	**Essential in ** ***S. cerivisiae***	**Comments**^1^
TRAPP I/II:			
Bet3 (193)	hBet3/TRAPPC3 (180)	+	Bet3 family^2^
Trs31 (283)	TRAPPC5 (188)	+	Bet3 family^2^
Trs33 (268)	TRAPPC6 A&B (173, 130)	--	Bet3 family^2^
Bet5 (159)	TRAPPC1/MUM2 (145)	+	Bet5 family^2^ Expression of Ag peptides in melanoma
Trs20 (175)	TRAPPC2/SEDL (140)	+	Bet5 family^2^ Associated w/bone formation & SEDT
Trs23 (219)	TRAPPC4/Synbindin (219)	+	Bet5 family^2^ Associated with postsynaptic structures
Trs85/Gsg1 (698)	--	--	Required for late sporulation, vt autophagy pathway in *S. cerivisiae*

TRAPP II:			
Trs120 (1289)	NIBP (1246)	+	21% identity in 377 match length; Implicated in NF-kappaB signaling, & neuro-degenerative disorders
Trs130 (1102)	TMEM1/EHOC1 (1259)	+	20% identity in 327 match length; Proposed similarity to sodium channels; Candidate for certain epilepsy and autoimmune disorders
Trs65/Kre11 (560)	--	--	Important for cell wall biogenesis in *S. cerivisiae*

^1 ^Information from Incyte Genomic and SGD Databases, 7/3/06

^2 ^Bet3 and Bet5 families was defined based on sequence and structure similarities [17, 52].

TRAPPII is a ~1000 kDa complex that contains three subunits in addition to those of TRAPPI: two large, essential subunits (>1000 amino acids), Trs120 and Trs130, and one small, non-essential subunit, Trs65 [15]. In yeast, Trs130 was implicated in late Golgi transport, whereas Trs120 was suggested to function in endosome-to-Golgi transport [15,18]. The conservation of the TRAPPII complex is less clear than that of TRAPPI [19]. Blast analyses show that the closest mammalian homologues of Trs120 and Trs130, NIBP and TMEM1, respectively, share a ~20% identity with the *S. cerivisiae *proteins over about a third of the protein (Table 1) as compared to 23–34% identity for five of the seven TRAPPI-specific proteins and 56% for Bet3 (Trs85 is not conserved, [16]). Only TMEM1 was shown to co-precipitate with the human TRAPP complex [20], and there is no functional evidence for a role for either of these proteins in protein trafficking. Instead, NIBP and TMEM1 were implicated in very different functions, NF-kappaB signaling and as a membrane channel, respectively [21-24].

There is no obvious mammalian ortholog for Trs65. However, current evidence supports a role for Trs65 in the *S. cerivisiae *TRAPPII complex. First, deletion of *TRS65 *is synthetic lethal with deletion of the non-essential TRAPPI subunit, *TRS33*. This synthetic lethality can be rescued by over-expression of Ypt31 [25,26]. In addition, Trs65/Kre11 was shown to function in cell-wall biogenesis [27], a process that is dependent on protein transport.

Because of the low sequence conservation by Blast analysis of the TRAPPII-specific subunits (Table 1), and the discrepancy between their suggested functions in fungal and mammalian cells, it is important to establish any evolutionary conservation more precisely. Here, we show a phylogenetic analysis of the TRAPPII-specific subunits. We found that nearly every fully sequenced eukaryotic proteome present in the NCBI non-redundant protein database (as for July 06) contains one Trs130 and one Trs120 homolog. We found three exceptions, which are noted in Results. In contrast, Trs65 is conserved among many, but not all, unicellular eukaryotes, and not in any fully sequenced multi-cellular proteome. The conservation of Trs120 and Trs130 is supported by conserved predicted secondary structure of whole proteins, and conserved domains that include highly conserved amino acids, which should also help in future functional analysis. In addition, our analysis suggests that neither Trs130 nor TMEM1 is a trans-membrane protein, arguing against the suggestion that TMEM1 functions as a membrane channel. Understanding the function of the human homologues is important, because they are candidates for several human disorders (Table 1).

## Results

### Phylogenetic trees of TRAPPII-specific subunits

**Figure 1:**
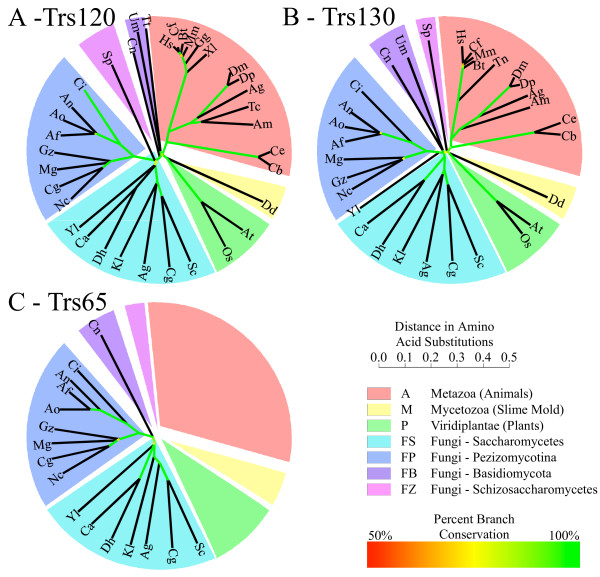
**Unrooted phylogenetic trees of TRAPPII-specific subunits**. A. Trs120; B. Trs130; C. Trs65. Colored pie sections demarcate groups noted in the key. Phylogenetic distances are shown in the scale bar. Confidence measurements from bootstrap analysis (1000 iterations) are shown by branch color (from 50%, red, to 100%, green). Organisms are abbreviated as detailed in Table S1.

We searched the non-redundant protein NCBI database on 07/2006 for sequences related to the three *S. cerivisiae *TRAPPII-specific subunits: Trs120, Trs130, and Trs65 (Table S1-A in Additional File 1). We identified 35 Trs120-related protein sequences and 31 TRS130-related protein sequences. We identified no more than one ortholog for each of these proteins in all examined genomes from fungi to vertebrates. In the 14 fully and 23 largely sequenced genomes, we always identified exactly one copy. We found sixteen Trs65-related sequences, also no more than one per genome, but only in fungi. Further, even some fully sequenced fungi (e.g., *Schizosaccharomyces pombe*) lack any recognizable ortholog of this protein (Figure 1 and Table S1-A in Additional File 1).

In addition to the sequences found in the NCBI database at the time of our search, we also looked at a 24 other largely completed genomes (including 19 additional fungi and 5 protists) that were at varying stages of assembly and annotation as of December 06. In spite of the incomplete nature of these projects, we still found Trs120 and Trs130 in 21 genomes, and Trs65 in 17 genomes. We show these results in Table S1-B (in Additional File 1).

There are a few interesting exceptions to our observations of the universality of Trs120- and Trs130-related sequences in eukaryotes. The three eukaryotic genomes that did not contain Trs120 and Trs130 are: the fungus *Encephalitozoon ecuniculi*, the Amoebazoan *Entamoeba histolytica *and the Archaeplastid *Cyanidioschyzon merolae*. It seems reasonable to suggest that the absence of Trs120 and Trs130 from the proteome of the first organism can be explained by the fact that it is an obligate intracellular parasite that might lack some essential cellular machinery. In agreement, three essential TRAPPI subunits are also missing in its genomes (Table S1-C in Additional File 1). *E, histolytica CMerolae*, however, are well-sequenced genomes, the latter is a red alga with a very small genome. Interestingly, the two genomes are also missing one essential TRAPPI subunit each (Table S1-C in Additional File 1). If the absence of TRAPP subunits in these organisms is confirmed, it might shed light on the evolution of the intra-cellular trafficking machinery.

The full-length protein sequences from the NCBI non-redundant (nr) database were used to construct un-rooted phylogenetic trees for each TRAPPII-specific subunit employing programs described in Methods. We used two different methods to construct the trees: PAUP's distance method, and PHYML's maximum likelihood (ML) method. Both methods yielded essentially identical topologies, except for branches near the center of the tree representing far-flung subunit-family members. Only branch lengths differed significantly between the two methods, with the ML method giving consistently ~4 fold longer branch lengths. Because we could estimate branch retention probabilities using PAUP, we show just the PAUP method results in Figure 1. The reliability of each branch position was assessed by distance-based bootstrap analysis and indicated by red (weak) to green (strong) colors. In almost all cases branches were maintained in almost all bootstrap trials, excepting only those close to the center of the tree, which reflect the details of ancient divergence between phylogenetic groups. The colored pie sections delineate groups of proteins in accord with tree branching and taxonomic relationships. There are seven basic groups: animals (A), plants (P), slime molds (M), and four fungal (F) groups Saccharomycetes (FS), Pezizimycotina (FP), Basidiomycota (FB), and Schizosaccharomycetes (FZ). Grouping in the tree corresponds in almost all cases to recognized taxonomic groupings [28]. The sole exception was *Yarrowia lipolytica *(FS_Yl), which is a Saccharomycete, but falls (weakly) with the Pezizomycotinas on the Trs130 tree.

### Multiple alignment analysis of TRAPPII-specific subunits

**Figure 2:**
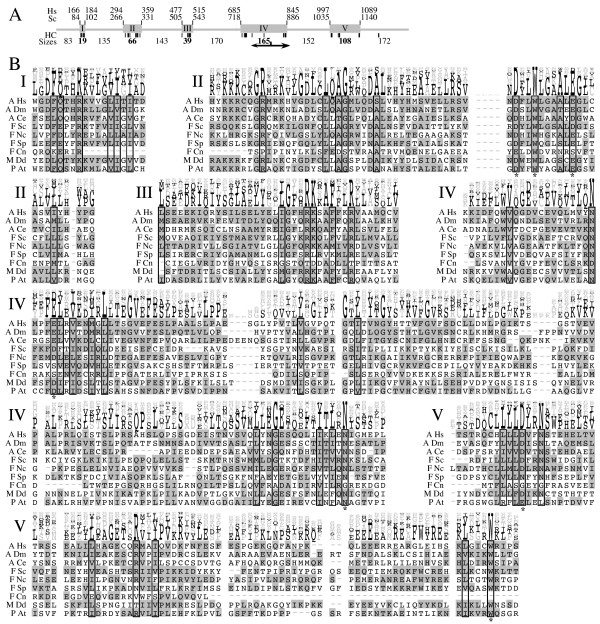
**Trs120 is conserved in almost all eukaryotes**. **A**. The domain structure of Trs120 is shown. Hs and Sc show amino acid coordinates in *H. sapiens *and *S. cerivisiae*, respectively. HC indicates highly-conserved amino acids described in Table S2. "Sizes" shows the median amino-acid lengths of all inter- and intra-domain lengths (intra-domain sizes are in bold). Arrows indicate a functional breakpoint. C-terminal truncation from this point results in a temperature-sensitive growth phenotype, while truncation N-terminal to this point is lethal. Actual sequences corresponding to these regions can be viewed at [50]. B. We show the multiple alignment of representative organisms for the sequences of each domain, I-V. Whole sequence alignment of all sequences from the NCBI nr database is shown in Figure S1. Organism abbreviations are as given in Table S1. For each position in the alignment, all amino acids belonging to the popular amino-acid grouping [42] best representative of the characters acids at that position, are marked in grey. Positions without sequence correspond to stretches in the alignment where one or two sequences contain long insertions that do not occur in the other sequences. Boxes were drawn to indicate the HC positions shown in A. To indicate the overall contribution of each of the amino acids at each position, we also show an Alscript [51] diagram above the multiple alignment. The height of each amino acid indicates its relative contribution to the alignment at that position, ignoring insertions (-). Amino acids corresponding to the most common amino acid grouping are written in black, while all other amino acids are written in gray.

**Figure 3:**
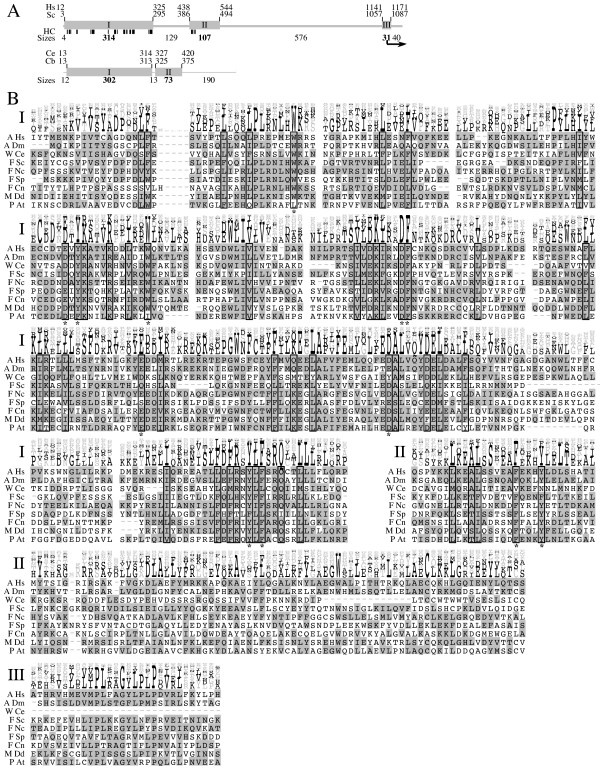
**Trs130 is conserved in almost all eukaryotes**. Same as Figure 2 except that A-bottom shows the domain structure for worm proteins, because these sequences have a deletion of domain III. Also, only a C-terminal-truncated temperature-sensitive mutant is shown. HC amino acids are detailed in Table S3. Whole sequence alignment of all sequences from the NCBI nr database is shown in Figure S2.

**Figure 4:**
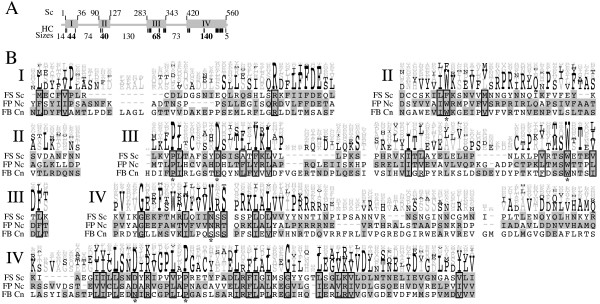
**Trs65 is conserved in fungi**. Same as Figure 2, HC amino acids are detailed in Table S4. Whole sequence alignment of all sequences from the NCBI nr database is shown in Figure S3.

Multiple alignments and the domain structure of the TRAPPII-specific subunits are shown in Figures 2, 3, 4 and Figures S1-S3 (see Additional File 1). Domains were defined by inspection of the raw alignment and Figures S1-S3. Highly conserved (HC) amino acids in the alignment (see Methods) are shown at the bottom of the diagram as short vertical lines, and are detailed in the corresponding supplementary tables (Tables S2-S4, respectively, see Additional File 1). There is a number of particularly interesting HC amino acids, including those that are invariant and rare (e.g., prolines) or invariant and potentially catalytic (e.g., aspartic or glutamic acids). These HC amino acids are indicated with asterisks in the multiple alignment and in the supplemental tables (Tables S1-S3, see Additional File 1). Finally, conserved domains, as indicated by alignment strength and HC amino acids are boxed (Figures S1-S3) and the alignment of these conserved domains from representative organisms is shown in Figures 2, 3, 4.

Multiple alignment analysis of Trs120-related proteins is shown in Figures 2 and S1. This figure shows five well-conserved domains across the length of Trs120, each characterized by high-quality sequence similarities and by the presence of HC amino acids in the alignment. These domains vary in size from 20 to 165 amino acids. Larger-scale sequence conservation and HC amino acids agree with one another: HC amino acids (detailed in Table S2) are found only in the otherwise conserved regions (except for a lone conserved glycine in a region of weak sequence conservation, C-terminal to domain TRS120-V.)

Trs120 is essential for *S. cerivisiae *cell viability. However, truncation of the C-terminal third of Trs120 in *S. cerivisiae *(amino acids 808 through 1289, see arrows in Figures 2B and S1), which includes part of conserved domain IV and all of domain V, results in a temperature-sensitive growth phenotype. Deletions further N-terminal of this point are lethal [18]. Therefore, we predict that domains I, II, and III and part of IV are important for the essential interactions of Trs120.

Multiple alignment analysis of Trs130-related proteins is shown in Figures 3 and S2. In this figure, the worms *Caenorhabditiselegans *and *Caenorhabditisbriggsiae *are slightly different in that they share a C-terminal deletion. Alignment and HC amino acids show three Trs130 domains ranging from 33–314 amino acids. We found no significant HC amino acids (detailed in Table S3) outside of these domains. Domains I and II contain many highly unusual HC amino acids. Consequently, we predict that these domains are critical for essential interactions or functions of Trs130.

Domain III is unlikely to be strongly important. It is shorter and contains no significant HC amino acids. Truncation C-terminal to the arrow shown in this domain in *S. cerivisiae *results in a temperature-sensitive growth phenotype [15,26]. While it is true that this domain might be important for the stability of the Trs130 protein, based on the low level of the protein in mutant cells [16], it might not be true for all organisms, since the two worm species share a C-terminal truncation of Trs130 (confirmed as described in Methods) that includes part of Trs130-II and all of Trs130-III.

Multiple alignments of Trs65-related proteins are shown in Figures 4 and S3. The protein is common for Saccharomyceta and Pezizomycotina subphyla (and at least one Basidiomycota), but not all fungi. Table S1 (see Additional File 1) shows that Trs65 also occurs in at least two other non-fungal unicellular eukaryotes. Since Trs65 is only found in some fungi, we used all three fungal groups shown in Figure 1 that contain Trs65 sequences. HC amino acids (detailed in Table S4) and alignment qualities show that these organisms share four conserved domains varying in size 40–140 amino acids. Although no mutagenesis data exists for these proteins, there are some highly unusual amino acids in domains II, III, and IV. Therefore we suggest that they are important for Trs65 function.

### The predicted secondary structure of Trs120 and Trs130 is conserved

**Figure 5:**
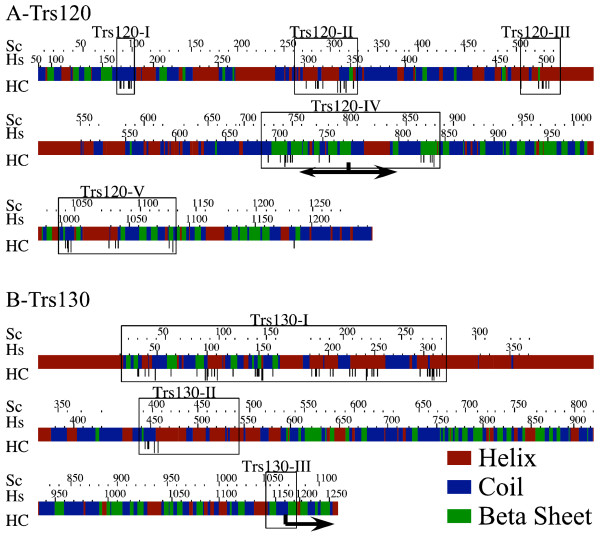
**Predicted secondary structure of Trs120 and Trs130 is conserved across eukaryotes**. A. Trs120; B. Trs130. Secondary structures were predicted by Prof [29] for all studied sequences and drawn with conserved boxes, HC amino acids, and mutagenesis-derived breakpoints. Secondary structures are predicted to be either helical (red), beta sheets (green), or coiled (blue). Similar data on a per-group basis is presented in Figure S4. Arrows indicate functional breakpoints. C-terminal truncation from the indicated points in both Trs120 and Trs130 results in a temperature-sensitive growth phenotype, while truncation N-terminal to this point in Trs120 is lethal.

We suspected that protein secondary structure was conserved in addition to its primary sequence. Since no crystal data is currently known for the three TRAPPII-specific subunits, we predicted their secondary structures using Prof [29]. This program employs multiple sequence alignments to predict the helical, beta-sheet, or coiled-coil nature of each position in the primary sequence of a group of proteins. We examined these predictions for all the Trs120, Trs130, and Trs65 proteins, using the clustal alignment employed to construct the trees and alignments in Figures 1, 2, 3, 4 for the input to Prof. Color-coded bars, representing the predicted secondary structures (helix-red; coil-blue; and beta sheet-green) are shown in Figures 5, S4 and S5, along with the conserved domains and HC amino acids described in Figures 2, 3, 4. To be sure that differences among evolutionary groups were in agreement with these results, we also chose three groups of species that contain a large number of sequenced proteins (6–13) and that are evolutionary far from each other: animals (A), and two fungi groups, Saccharomycetes (FS), and Pezizomycotina (FP), and show these results in Figures S4 and S5.

Surprisingly, the predicted secondary structure of Trs120 and Trs130 is highly conserved not only in domains that are conserved for primary structure, but also along the whole sequence of these proteins, even where there is low sequence identity between the groups (Figures 5 and S4). Therefore, the predicted secondary structure of these two proteins is conserved better than their primary structure. Similar analysis of Trs65 among the Saccharomycetes and Pezizomycotina fungal groups shows conservation of predicted secondary structure among members of the same group. However, these predictions do not agree well between the two subphyla (Figure S5). This suggests that unlike Trs120 and Trs130, the interactions, and perhaps the specific functions, of Trs65 are not well conserved even among all fungi.

For the three protein families, we searched all studied sequences (from all organisms) for potential structural motifs using SMART [30,31]. We found some isolated SMART hits, but none were consistent across a whole group (i.e., all animals, all of a fungal subphylum, etc). Notably, none of the isolated hits overlapped the conserved domains shown in Figs 2, 3, 4. Portions of the conserved domains of TRAPPII-specific subunits seem good prospects for inclusion in future releases of such motif-finding engines.

### Trs130 is a membrane-associated protein

Based on sequence analysis, the *S. cerevisiae *Trs130 and its human homolog were suggested in 1997 to contain trans-membrane domains (TMDs). In fact, the human homologue was named TMEM1, for Trans-Membrane Epilepsy Myoclonus [23]. However, the yeast TRAPP complex is only membrane associated, because it can be removed from membranes by salt but not by detergent [32,33], even though this was not shown specifically for Trs130.

**Figure 6:**
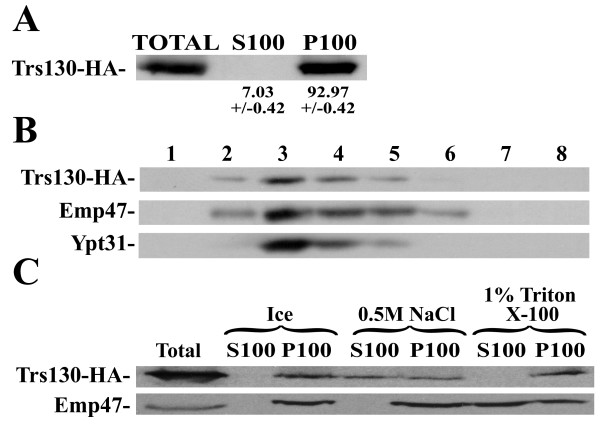
**The *S. cereviciae *Trs130 behaves as a membrane-associated, not trans-membrane, protein**. A. Trs130 is found in the P100 fraction of cell lysates. Lysates (from 65 OD_600 _cells) from wild type (NSY991) cells expressing Trs130-HA were fractionated by 100,000 × g centrifugation to yield supernatant, S100, and pellet, P100, fractions. The fractions (from 6.5 OD_600 _cells) were tested by immuno-blot analysis using anti-HA antibody. B. Trs130 floats with membranes on OptiPrep gradients. The P100 fraction (from 50 OD_600 _cells) from panel A, was loaded on an OptiPrep gradient. After centrifugation, the fractions were tested by immuno-blot analysis using anti-HA, anti-Ypt31 and anti-EMP47 antibodies. Trs130-HA fractionates with two other membrane proteins, Ypt31 and EMP47. C. Trs130 can be extracted from the P100 fraction by salt, but not by detergent. Resuspended P100 fractions (100 μg) were incubated with 0.5 M NaCl or 1% Triton X-100, and subjected to centrifugation. 50 μg were examined by immuno-blot analysis as described in panel A. Emp47 serves as a control for a trans-membrane protein that can be extracted by detergent, but not by salt. Bands were quantified and percent distribution in S100 and P100 is shown. Results in this figure are representative of three independent experiments.

Because the *S. cereviciae *Trs130 contains two putative trans-membrane domains [34], we wished to determine whether this protein is a trans-membrane or a membrane-associated protein. Trs130 isolated from yeast cell lysates is found in the P100 (pellet of 100,000 × g) particulate fraction of cell lysates (Figure 6, panel A), which floats with membranes on an OptiPrep gradient (Figure 6, panel B). If Trs130 were a trans-membrane protein, it could be extracted from the membrane by detergent, but not by salt. However, treatment of the P100 fraction with salt, and not with detergent, yields soluble Trs130 (Figure 6, panel C). Therefore, the yeast Trs130 behaves as a membrane-associated protein, and not as a trans-membrane protein. These results imply that the observed hydrophobic domains do not function as TMDs, but rather are important for the proper folding or interactions of Trs130.

This idea that TMEM1 and Trs130 are trans-membrane proteins was based on sequence analysis, so we revisited this sequence analysis for the human TMEM1. Previously, sequence analysis of the human TMEM1 suggested that the protein contained two (using SOSUI, [23]) or multiple [24] trans-membrane domains (TMDs). We tested the human TMEM1 sequence for putative TMDs using SOSUI [35] and SMART [30] on 6/06, and no TMDs were detected by these programs. Note that SMART uses TMHMM2.0 to predict TMDs, a method which has been shown to be among the most accurate at such tasks [36]. We suspect that the TMD-detecting algorithms have become more discriminating in the intervening years and suggest that, like the *S. cerivisiae *Trs130, the human protein is a membrane-associated protein, not a trans-membrane protein as suggested by its original name.

## Discussion

We show here that the TRAPPII-specific subunits, Trs120 and Trs130, are conserved from fungi to animals. This conclusion is based on sequence and predicted secondary structure analyses. Multiple alignments analysis reveals conserved domains with highly conserved amino acids in each protein family. Predicted secondary structure analysis reveals conservation in the clustal/HC-derived domains as well as in the intervening sequences. The conservation of the predicted secondary structure and the fact that the secondary structure of these proteins is conserved better than their primary structure supports the analysis presented here. This suggests that TRAPPII, like TRAPPI, is a conserved complex, and that the function of both TRAPP complexes, in the regulation of trafficking through the Golgi apparatus, is also conserved.

In contrast, the non-essential subunit, Trs65, is conserved only between some fungi. The Trs65-related proteins also share conserved domains with highly conserved amino acids. However, their secondary structures as predicted by Prof are not highly conserved between two evolutionary distant fungal groups, Saccharomycetes (FS) and Pezizomycotina (FP). The fact that Trs65 is not essential for viability in *S. cerivisiae*, and is only weakly conserved among some unicellular eukaryotes, suggests that Trs65 has a fungi-specific function. Indeed, it was implicated in *S. cerivisiae *in cell-wall biogenesis [27]. Alternatively, other organisms may have a functionally similar subunit that does not share sequence similarity with Trs65.

The *S. cerivisiae *TRAPP complexes act as activators, GEFs, for the Golgi Ypts [13,16,32]. In general, Ypt/Rab GEFs are large protein complexes [37], and there is no similarity between GEFs for different Ypt/Rab paralogs. This suggests the recruitment of Ypt/Rab GEFs from divergent families. The reason for this might stem from the divergence of factors that regulate these GEFs, about which, very little is currently known. Thus, the diversity of the Ypt/Rab GEFs suggests that GEFs that function in different cellular compartments are regulated by diverse upstream factors. Alternatively, Ypt/Rab GEFs might have diverged beyond recognition, which is hard to believe in light of the conservation of the whole trafficking machinery.

Unlike the Ypt/Rabs, for which the number between fungi and mammalian cells increases by about seven fold, the number of TRAPPI and TRAPPII subunits per genome remains constant through evolution (except for two mammalian paralogs of Trs33, A and B, see Table 1). This ratio of Rab/GEF suggests that in mammalian cells TRAPP acts as a GEF for more Rabs than in *S. cerivisiae*, perhaps for whole groups of Ypt1/Rab1- and Ypt31/Rab11-related proteins [6]. Alternatively, more animal GEFs evolved during evolution that do not share sequence similarity with the fungal GEFs.

### Domains and functions

The conservation of the primary and the secondary structures of Trs120 and Trs130 suggests that they cooperatively execute an important cellular function. We propose that conservation of secondary structure of the well-conserved domains (defined by sequence conservation and the presence of highly conserved amino acids) probably reflects conservation of catalytic functions and/or protein-protein interactions of these domains. The latter are known to place constraints on the divergence of residues in contact surfaces. In contrast, conservation of the secondary structure of the intervening sequences might be required for the three-dimensional orientation of the well-conserved domains.

**Figure 7:**
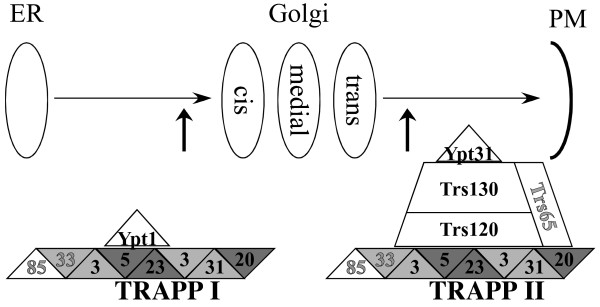
**Model for the conserved structure and function of the TRAPPI and TRAPPII complexes**. TRAPP complexes mediate exocytic trafficking from the ER, through the Golgi, to the plasma membrane. The TRAPPI complex (left) is shown as a quadrilateral with subunits indicated as triangular sections. Subunits are 3-Bet3, 5-Bet5, 20-Trs20, 23-Trs23, 31-Trs31, 33-Trs33. The Bet3 family (3, 31, 33), are shown as identically light gray triangular sections. The Bet5 family (5,20,23) are shown as identically dark gray triangular sections. The displayed arrangement of components is derived from Kim et al., 2006 [17]. The TRAPPII complex (right) includes all the TRAPPI components plus the three TRAPPII-specific subunits shown in the trapezoidal shape above TRAPPI. In both complexes, subunits essential for viability are labeled in black letters, while non-essential subunits are shown in gray letters. Functionally, TRAPPI is the GEF for Ypt1, the GTPase (GTPases are shown as triangles) that regulates entry into the Golgi, whereas TRAPPII is the GEF for Ypt31/32, the GTPases that regulate exit from the Golgi [16]. All shapes have areas proportional to the corresponding amino acid lengths, except for Trs85, which is larger than shown.

We have previously suggested a role for the TRAPP complex in the coordination of entry into and exit from the Golgi [14,16], and for the TRAPPII-specific subunits, in the specificity switch of the GEF activity of TRAPP from Ypt1 to Ypt 31/32 ([16], and Figure 7). Based on our functional analysis of the *S. cerivisiae *Trs120 and Trs130, we propose a number of protein-protein interactions for the two essential TRAPPII-specific subunits. We predict at least two protein-protein interactions for Trs120, with Trs130 and with TRAPPI [16]. The four Trs120 conserved domains (I-III, and part of IV), which are essential for *S. cerevisiae*, are candidates for these interactions. For Trs130, we predict at least two protein-protein interactions and a possible catalytic role in the Ypt31 GEF activity of TRAPPII [16]. The two conserved Trs130 domains that are essential for *S. cerevisiae *viability, I and II, are candidates for these functions. The identification of Trs120- and Trs130-conserved domains and HC amino acids will allow future functional analysis of the separate domains of these large proteins.

### Proposed roles for the mammalian Trs120- and Trs130-related proteins

We suggest that the role of the two TRAPP complexes as Ypt/Rab GEFs and their coordination function are conserved from fungi to animals. However, the mammalian orthologs of Trs120 and Trs130 were implicated in other cellular processes. The mammalian Trs120-related protein, NIBP was implicated in NF-kappaB signaling [21]. While the suggested functions of the fungal and mammalian orthologs of Trs120 do not overlap, they do not contradict each other, and it is possible that trafficking through the Golgi in mammalian cells is regulated by NF-kappaB signaling.

In contrast, the mammalian Trs130-related protein, TMEM1 (for Trans Membrane Epilepsy Myoclonus), was implicated in a function distinct from protein trafficking. While the *S. cerevisiae *TRAPP, including Trs130, is a membrane associated complex, TMEM1 was suggested to be a trans-membrane protein and to function as a membrane channel [23,24]. Our analyses suggest that both the *S. cereviciae *Trs130 (by membrane-extraction analysis) and the human TMEM1 (by sequence analysis) are not trans-membrane proteins. Based on our alignment and predicted secondary structure analyses, we propose that the function of Trs130 as a specificity switch for TRAPP's GEF activity is conserved. These ideas can now be tested.

### Trs120 and Trs130 and human disorders

Regulation of critical steps of the exocytic pathway is bound to be crucial in understanding the basis of every disease that is connected with secretion of a substance or presentation of a receptor on the plasma membrane. Indeed, several TRAPPI subunits were implicated in human diseases. For example, a missense mutation in human Bet5/TRAPPC1 results in the expression of antigenic peptides in melanoma [38]; and the SEDL/Trs20 gene is responsible for SEDT, an Xlinked skeletal disorder [39]. The two mammalian orthologs of Trs120 and Trs130 were also implicated in human disorders: NIBP, the Trs120 ortholog, was implicated in neurogenerative disorders, based on its connection to NF-kappaB signaling [21]. The Trs130 ortholog, TMEM1, is a candidate for several human disorders, including certain types of epilepsy, autoimmune, and holoprosencephaly disorders, based on genetic linkage studies mapping genes responsible for these disorders to a chromosome region that includes TMEM1 [23,24,40]. Therefore, resolving the probably conserved function of the mammalian NIBP and TMEM1 proteins should help elucidate the basis for these disorders.

## Conclusion

TRAPP is a modular protein complex that regulates entry into and exit from the Golgi, which is a cellular compartment central to multiple trafficking pathways. TRAPPI, the complex that functions in the entry to the Golgi, is highly conserved. We have recently shown that the two yeast essential TRAPPII-specific subunits are required for changing the activity of TRAPPI to that of TRAPPII [16]. Here, we explored the conservation of the TRAPPII-specific subunits. We show that the primary and secondary structures of the two essential subunits, Trs120 and Trs130, are conserved from yeast to man, whereas the non-essential Trs65 is conserved only among some unicellular eukaryotes. We suggest that the role of the human orthologs of Trs120 and Trs130, NIBP and TMEM1, respectively, in the regulation of intra-cellular trafficking is also conserved. Conserved domains and highly conserved amino acids in these domains should help future functional studies of these proteins in higher eukaryotes.

## Methods

### Identification of TRAPPII-specific subunit sequences

We searched the non-redundant protein database from NCBI on 7/06 [41]. Trs120-containing proteins were identified by iteratively performing BLAST analysis of this database with the *S. cerivisiae *Trs120 sequence with an expectation value of 10^-5^. Hits from the first and subsequent rounds of BLAST analysis were used to perform additional analyses until no more sequences were discovered. An identical procedure was used for the identification of Trs130- and Trs65-containing proteins, again using the *S. cerivisiae *sequences as seeds.

Table S1-A shows all identified sequences including abbreviated names used in all figures, protein sequence length, accession numbers, and annotations from the NCBI database. Full-length versions of each sequence were identified for all further work, discarding truncations or deletions. We used these Trs120, Trs130, and Trs65 proteins to search 24 additional, largely completed genomes in various stages of completion and annotation. We searched in both called proteins sequences and in the raw genome using tblastn. The sequence source and the presence or absence of Trs120, Trs130, and Trs65 in these genomes is shown in Table S1-B.

For Trs130, the two worm sequences, *C. briggsae *and *C. elegans*, seemed to be truncated relative to the remaining Trs130-containing proteins. We searched both completed genomes in all six reading frames using the universal translation table for sequences corresponding to the C-terminal remainder of any of the other animal proteins. We found no significant blast hits.

### Construction of the phylogenetic trees

The identified Trs120, Trs130, and Trs65 proteins were aligned by clustal [42], using the default settings for slow/accurate alignments (gap penalty of 10, gap extension cost of 0.2, 30% delay for divergent sequences, 4 space gap separation distance, without end gap separation, with residue-specific penalties, and using the Gonnet series protein weight matrix). The aligned sequences were manually trimmed on the N- and C-terminal ends to remove weak or ambiguous alignments. Phylogenetic analysis was performed using PAUP 4.0b10 [43] and PHYML [44]. Distance trees were created using the heuristic distance search for optimal trees by PAUP and the maximum likelihood method by PHYML. The PAUP tree was started with neighbor joining, and branch swapping used the TBR algorithm. We used default settings for the ML method (JTT substitution model, 1 substitution rate category, optimization on for branch lengths and topology). To estimate the reliability of the PAUP tree, bootstrap analysis was performed with 1000 replicates of full heuristic searches, using the same weighting parameters employed in the initial analysis. The trees were drawn by TreeView version 1.6.6 [45] and manually modified in a general-purpose graphics editor. Branches were color coded from red to green to reflect their persistence in bootstrap analysis in 50–100% of sampled trees. We constructed unrooted trees for the TRAPPII-specific subunits because we could not find any sequences that are related to them, even at an extremely lenient blast cutoff (e<10^-2^), and are unambiguously diverged from all eukaryotes, e.g., from archaeal or bacterial proteomes.

### Domain structure and identification of Highly Conserved (HC) amino acids

Domains were defined by inspection of the raw clustal files and of the graphical multiple alignments shown in Figures S1-S3. Domains were consistently present in all sequences in all groups of organisms examined.

We examined the clustal-generated alignments for highly-conserved (HC) amino acids, using popular amino acid groupings [42]. Alignment positions were considered HC if more than 90% of the residues belonged to an amino acid group. Since some groups (MILV, MILF, and SAT) are quite common, we discarded these positions if they were more than 20 amino acids away from any other HC position.

### Prediction of secondary structures

We predicted sequence secondary structures using Prof [29], a secondary structure predictor that uses multiple alignments to inform its prediction about the helical, beta-sheet, or coiled nature of each position in the primary sequence. We used the same clustal-generated alignment used for phylogenetic trees and annotated alignments for Prof analysis. To be sure that its results were consistent, we gave Prof either all the aligned sequences (ALL) just those from animals (A), just those from Saccharomycetes (FS), or just those from Pezizomycotina (FP). We wrote a custom perl program to draw these predicted secondary structures as red (helix), green (beta-sheet) or blue (coiled) bars alongside HC and domain annotations.

### Reagents

The following yeast strain was used in this study: wild type, NSY991 (VSY459; MATa *leu2-3,112 his3-200 trp1-901 lys2-801 suc2-9 ura3-52 TRS130-HA:HIS3MX6*) [26]. Antibodies used in this study: Mouse monoclonal Anti-HA (clone12CA5, Roche); affinity purified rabbit anti-Ypt31 [9]; rabbit anti-EMP47 (gift from H. Riezman, [46]); and horseraddish Peroxidase linked Anti-rabbit and anti-mouse IgG (Amersham Biosciences). All chemical reagents were purchased from Sigma (St. Louis, MO), unless otherwise noted. Iodixanol density gradients, Optiprep™, were purchased from Axis-Shield PoC AS (Oslo, Norway).

### Preparation of cell lysates and protein analyses

Yeast cells were grown in rich (YPD) medium [47]. Yeast cell extracts were prepared as previously described [48]. Cell breakage buffers were supplemented with an EDTA-free protease inhibitor cocktail (Roche Diagnostics, Indianapolis, IN). Protein concentrations were determined by BioRad protein assay (BioRad). 10 μg of yeast whole-cell lysates were loaded on 7.5–10% SDS-PAGE. Gels were run, and proteins were transferred to PVDF membranes and subjected to immuno-blot analysis. Quantification of protein bands was done using the AlphaEase FC and Alpha-Imager (Alpha Innotech Corporation).

### Membrane attachment analysis

Cell fractionation: Yeast cell lysates were prepared as described above with the following alterations: Frozen cell pellets (22–25 OD_600 _units) were resuspended and broken in 100 μl spheroplast buffer [49] by vortexing 3 times for 2 minutes each at 4°C. 100 μl of spheroplast buffer was added and the supernatant was separated from cell debris as previously described [48]. 100 μl of the supernatant was saved to be used as total cell lysates and the other 100 μl was taken for further centrifugation at 100,000 × g for 30 minutes at 4°C. The supernatant (S100) was separated from the pellet (P100). P100 was resuspended in spheroplast buffer to the same volume as S100. 75 μg total cell lysates and an equivalent of 1.5 OD_600 _units for P100 and S100 were subjected to immuno-blot analysis.

Iodixanol Density Gradient: Resuspended P100 fractions were analyzed on 30% iodixanol density gradients (Optiprep™, Axis-Shield PoC AS) according to the protocol provided by the company. Fractions were analyzed by immuno-blot analysis.

Membrane Extraction: P100 was resuspended in 100 μl B88 alone, or supplemented with 1% Triton X-100, or with 0.5 M NaCl as previously described [32]. Samples were centrifuged at 100,000 × g and subjected to immuno-blot analysis.

## Abbreviations

PM- Plasma Membrane; ER- Endoplasmic reticulum; GTP-Guanine Tri-Phosphate; GEF- Guanine nucleotide Exchange Factor; nr- Non Redundant; HC- Highly Conserved; TMD- Trans Membrane Domain.

## Authors' contributions

RC performed all sequence analyses, SHC and EY carried the protein analyses, NS with the help of the other authors, was involved in project planning, data analysis, and writing. All authors read and approved the final manuscript.

## Supplementary Material

Additional File 1 Supplementary Methods, Figures and Tables. Supplementary Figures S1-S5 show whole-sequence multiple alignments of Trs120, Trs130, and Trs65 proteins as well as predicted secondary structures of these subunits by taxonomic group. Supplementary Tables S1-S4 detail TRAPP II-specific subunits found in each genome and the highly conserved (HC) amino acids found in each subunit. Supplementary Methods applying to these supplementary data are described. Click here for file
