# Supplementary material for: Conservation of the TRAPPII-specific subunits of a Ypt/Rab exchanger complex
Source: BMC Evol Biol. 2007 Feb 2;7:12. doi: 10.1186/1471-2148-7-12 (PMC1803778; doi:10.1186/1471-2148-7-12)
Supplement: Additional File 1 — Supplementary Methods, Figures and Tables. Supplementary Figures S1-S5 show whole-sequence multiple alignments of Trs120, Trs130, and Trs65 proteins as well as predicted secondary structures of these subunits by taxonomic group. Supplementary Tables S1-S4 detail TRAPP II-specific subunits found in each genome and the highly conserved (HC) amino acids found in each subunit. Supplementary Methods applying to these supplementary data are described. [file 1471-2148-7-12-S1.pdf]

Additional File for "*Conservation of the TRAPP II-specific subunits of a Ypt/Rab exchanger complex*"

Title: Supplementary Methods, Figures, and Tables.

Methods

Multiple alignments within and across groups. Alignment illustrations in Figures S1-S3 were based on the clustal alignments described in the text and generated by a custom perl script to reflect comparisons of the qualities of alignments between and within groups of Trs120- Trs130- and Trs65-family proteins. All groups were assigned colors used in Figure 1. For a given group of proteins, the alignment was broken into segments according to cross-family alignments (e.g., a run of just fungal alignments, a run of worm and other animal alignment, etc). For regions with no alignment in at least half of the members of the group, the region is left white.

For each region, both intra- and inter-group similarity scores were computed. Intra-group scores were the average PAM250 (ALTSCHUL *et al.* 1997) value, averaged over all possible pairings between members of the group. Inter-group similarities were computed as the average PAM250 score of each possible pair consisting of one member of each of the two groups. Each comparison generated a bar colored according to the primary hue of the target group but with an intensity reflecting the quality of the alignment. PAM250 scores of less than 0 are drawn in black. Positive scores are drawn in the target group's hue but with a saturation scaled from 0 to 100% for PAM250 scores of 0 to 3, respectively.

Figures

In Figures S1-S3, proteins are arranged by groups (as in (COX *et al.* 2004)) identified by the phylogenetic tree analysis in Figure 1 of the main text: A-Animals, W-Worms (for S2 only), M-Mold, P-Plants, and F-Fungi.

The alignment shows each group by a different color and row. Within-group conservation appears as a horizontal bar of the same color as the group. Color intensities indicate average PAM scores between all members of the group, with black indicating no sequence similarity (0 or less) to bright colors indicating strong sequence similarity. Cross-group conservation appears as horizontal bars in each row, in the color of the other group; varying in intensity according to the strength of the average conservation between members of the two groups. Where no within-group conservation is present or where multiple alignments between groups require the addition of insertions, the figure shows a gap. Amino acids numbering is shown at the top for the *S. cerevisiae* and *H. sapiens* sequences.

Boxes in these figures mark areas of large cross-group sequence similarity. HC amino acids are groups of highly-conserved amino acids shown as short vertical lines at the bottom of the boxes and detailed in Table S2-S4. Particularly interesting HC amino acids are indicated in the figure as long vertical lines, and marked with asterisks in the table. Actual sequences corresponding to these regions can be viewed at http://www.uic.edu/~nava/papers/TRAPPII.

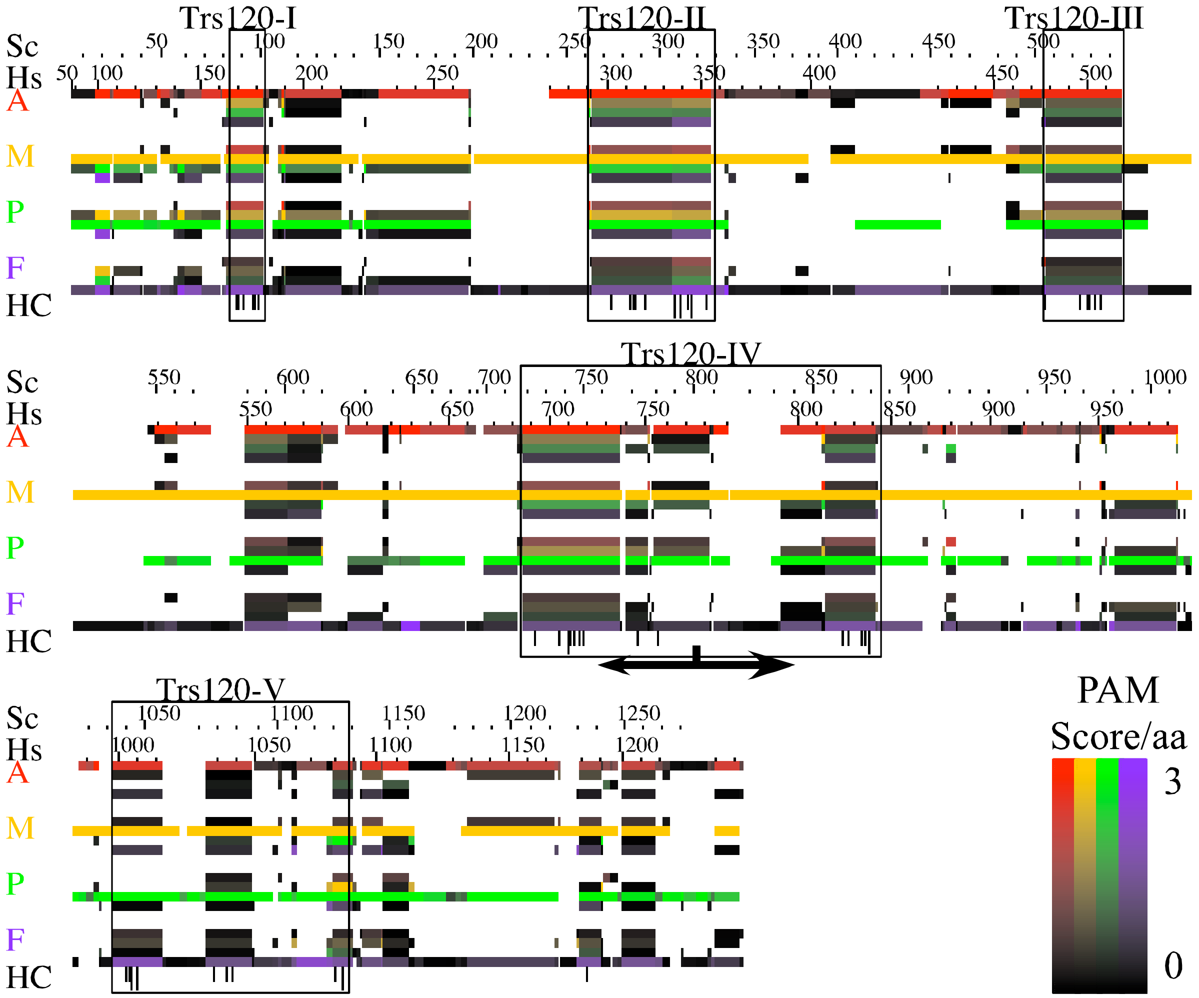

Figure S1: **Multiple alignments of whole Trs120 proteins from all genomes present in the NCBI nr database**. As described above except as follows: Although Tetrahymena thermophila (a member of the Alveolata lineage) does have a conserved Trs120 sequence, it is widely diverged from the other sequences, even in HC amino acids, as seen in Table S2. Consequently, we dropped T. thermophila when constructing this figure. Arrows indicate a functional breakpoint. C-terminal truncation from this point results in a temperature-sensitive growth phenotype, while truncation N-terminal to this point is lethal.

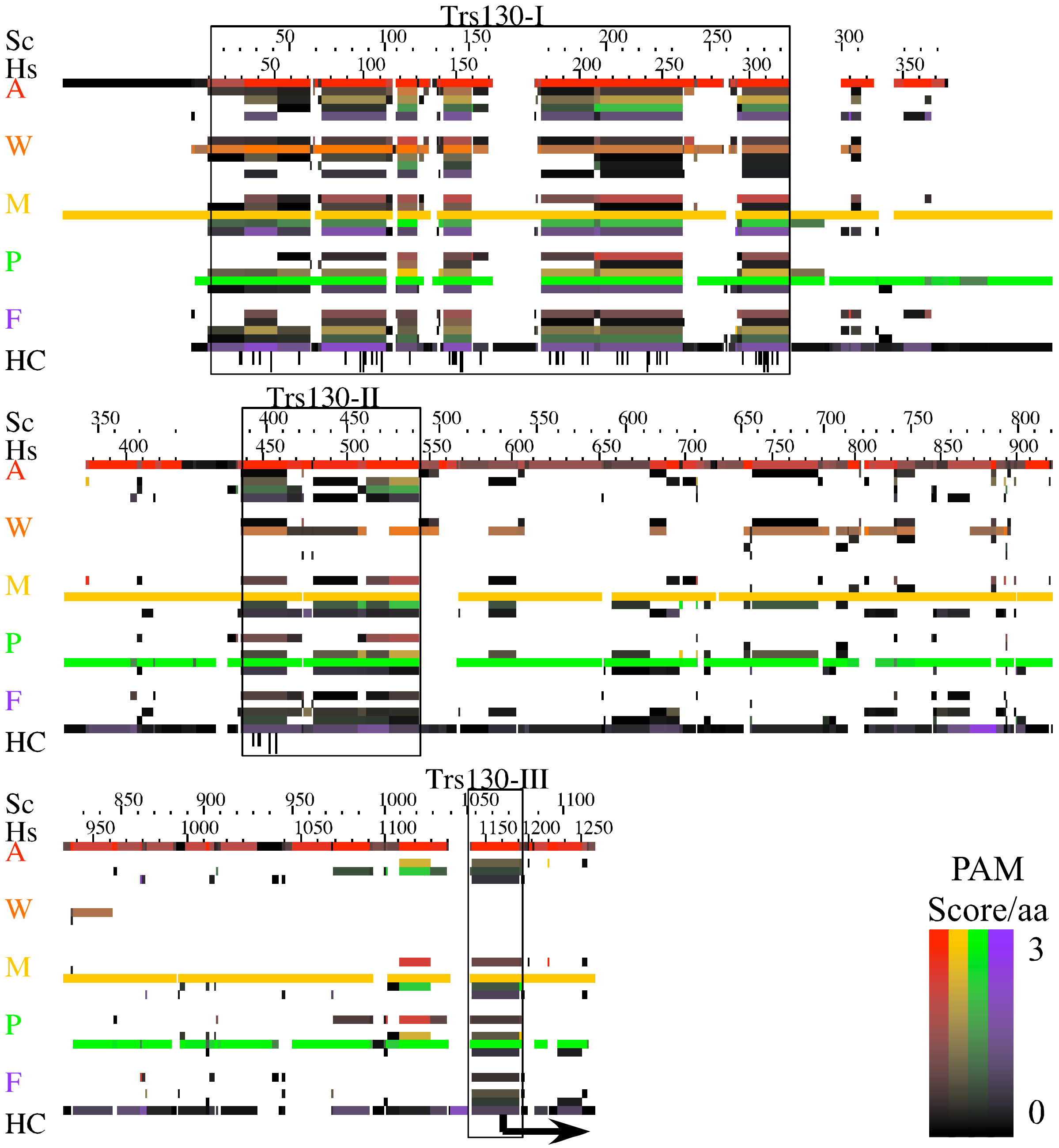

Figure S2: **Multiple alignments of whole Trs130 proteins from all genomes present in the NCBI nr database**. As described above, except that the arrow indicates that truncation C-terminal to this point confers a temperature-sensitive growth phenotype.

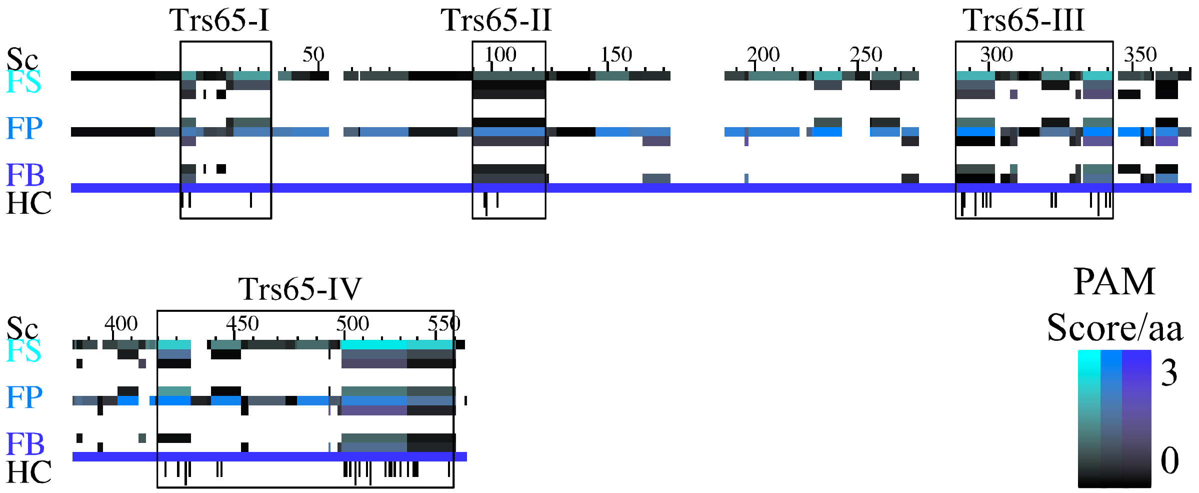

Figure S3: **Multiple alignments of whole Trs65 proteins from all genomes present in the NCBI nr database**. As described above, except that there are only three groups: FS-Saccharomycetes, FP-Pezizomycotina, FB-Basidomycota.

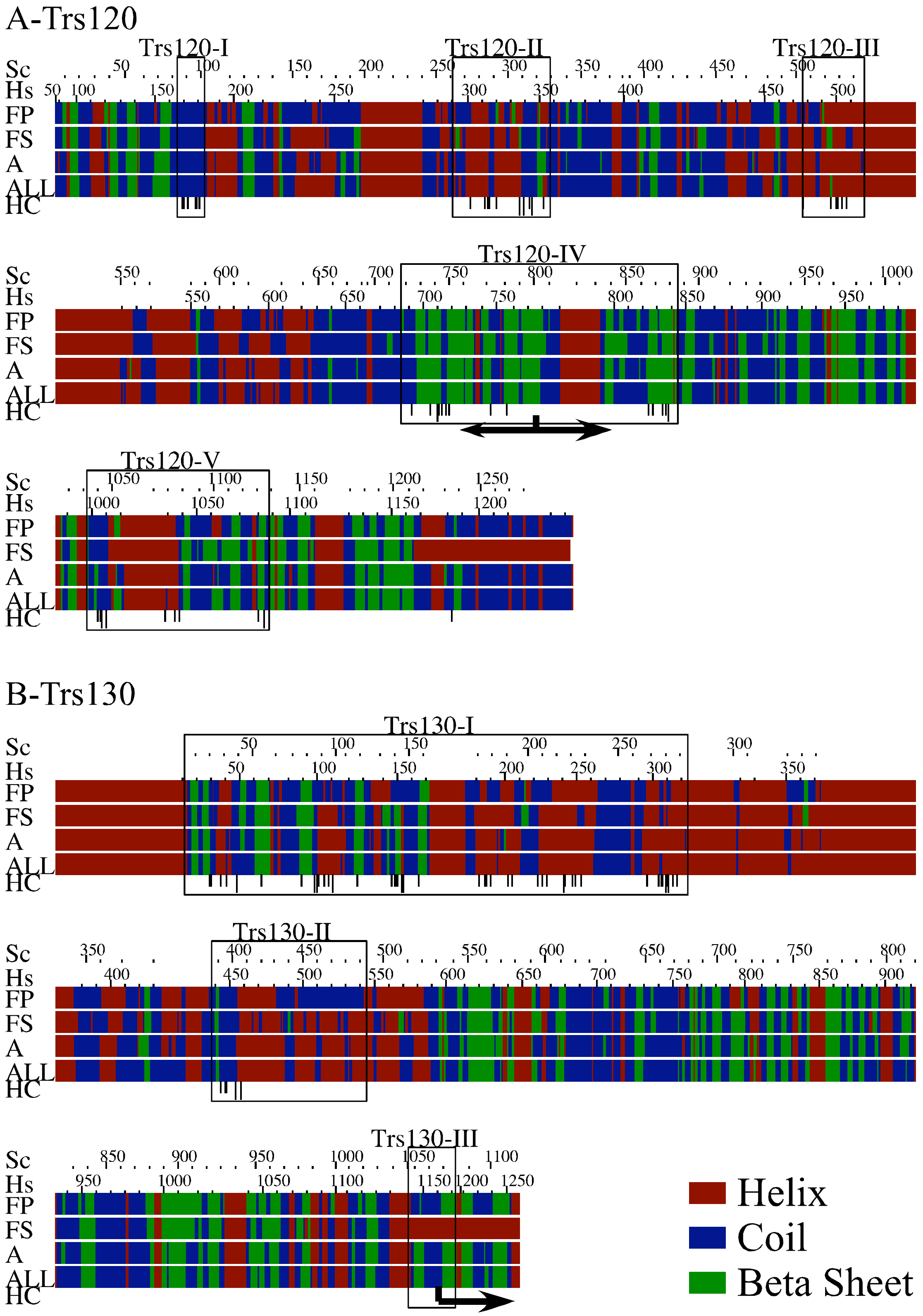

Figure S4: **Predicted secondary structure of Trs120, Trs130 proteins by groups**. A. Trs120; B. Trs130. Secondary structures were predicted by Prof (SODING 2005) for either FP, FS, A groups, or for all studied sequences (bottom bar), and drawn with conserved boxes, HC amino acids, and mutagenesis-derived breakpoints shown in Figures 5. Secondary structures are predicted to be either helical (red), beta sheets (green), or coiled (blue).

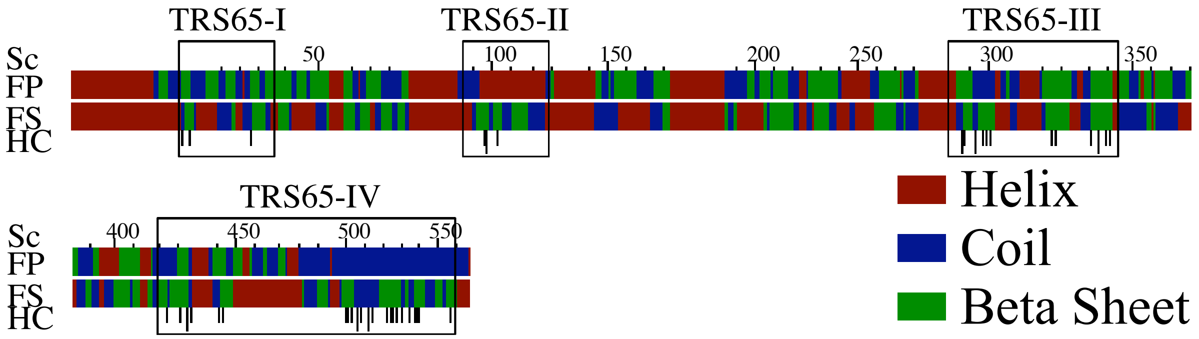

Figure S5: Predicted secondary structure of Trs65 proteins by groups. Same as in Figure S4.

Table S1: TRAPP II-specific subunits found in each genome

A: Genomes in the NCBI NR Database

			TRS120		TRS130		TRS65	
**Name[1]**	**Species**	**Status[2]**	**GI[3]**	**L[4]**	**GI[3]**	**L[4]**	**GI[3]**	**L[4]**
A_Ag	*Anopheles gambiae*	A	55244096	1300	55246262	1149	NF	
A_Am	*Apis mellifera*	A	66522062	1188	66500891	1174	NF	
A_Bt	*Bos taurus*	A	79158881	1138	76608304	1302	NF	
A_Cf	*Canis familiaris*	A	73974771	1147	74001521	1250	NF	
A_Dm	*Drosophila melanogaster*	C	24585462	1320	21428600	1145	NF	
A_Dp	*Drosophila pseudoobscura*	A	54645195	1311	54639473	1142	NF	
A_Dr	*Danio rerio*	I	P		NF		NF	
A_Gg	*Gallus gallus*	A	50731934	1758	P		NF	
A_Hs	*Homo sapiens*	C	34536287	1246	1778033	1259	NF	
A_Mm	*Mus musculus*	C	74140270	1139	82933646	1258	NF	
A_Pt	*Pan troglodytes*	A	P		P		NF	
A_Rn	*Rattus norvegicus*	A	P	1912	P		NF	
A_Sj	*Schistosoma japonicum*	I	NF		P		NF	
A_Sp	*Strongylocentrotus purpuratus*	I	P		P		NF	
A_Tc	*Tribolium castaneum*	A	91089065	1188	P		NF	
A_Tn	*Tetraodon nigroviridis*	A	P	885	47212555	1316	NF	
A_Xl	*Xenopus laevis*	I	38014735	1151	NF		NF	
A_Ce[5]	*Caenorhabditis elegans*	C	3874766	1224	48059937	611	NF	
A_Cb[5]	*Caenorhabditis briggsae*	I	39594746	1331	39589369	562	NF	
M_Dd	*Dictyostelium discoideum*	A	66820723	1464	66822839	1442	NF	
P_At	*Arabidopsis thaliana*	C	15238938	1186	8885587	1280	NF	
P_Os	*Oryza sativa*	C	32479981	1193	77556742	1245	NF	
FS_Ag	*Ashbya gossypii*	C	44980551	1198	44984022	1082	44986340	454
FS_Ca	*Candida albicans*	A	68473121	1274	68478822	1264	68480216	580
FS_Cg	*Candida glabrata*	C	50287319	1258	50287425	1096	50290401	506
FS_Dh	*Debaryomyces hansenii*	C	50426411	1460	50421807	1215	50422163	613
FS_Kl	*Kluyveromyces lactis*	C	50302331	1210	50305115	1066	50311427	427
FS_Sc	*Saccharomyces cerevisiae*	C	6320615	1289	6323874	1102	6321605	560
FS_Yl	*Yarrowia lipolytica*	C	50550737	1097	50547529	1129	50546635	608
FP_Af	*Aspergillus fumigatus*	A	70984150	1423	42820660	1451	70995277	549
FP_An	*Aspergillus nidulans*	A	67540726	1418	67517712	1433	67521578	529
FP_Ao	*Aspergillus oryzae*	I	83771685	1489	83766626	1424	83774065	584
FP_Cg	*Chaetomium globosum*	A	88179058	1442	NF		88185104	570
FP_Ci	*Coccidioides immitis*	I	90307727	1500	90301762	1572	90298080	562
FP_Gz	*Gibberella zeae*	A	46123723	1780	46121351	1514	46125443	626
FP_Mg	*Magnaporthe grisea*	A	39944090	1438	39967219	2016	39945238	655
FP_Nc	*Neurospora crassa*	A	85100072	1454	39967219	1631	85111300	702
FB_Cn	*Cryptococcus neoformans*	C	57226305	633	50255463	1173	50258075	812
FB_Um	*Ustilago maydis*	A	71020337	3893	71023513	1434	NF	
FZ_Sp	*Schizosaccharomyces pombe*	C	2330869	1210	3581914	1150	NF	
Al_Tt	*Tetrahymena thermophila*	I	89291072	1771	NF		NF	

1- Abbreviated name used in the figures and tables. 2- Status of the each organism's genome sequencing project as of 9/29/06 according to http://www.ncbi.nlm.nih.gov/genomes/leuks.cgi, where genomes marked with C are completed, those with A have extensive assembly, and those with I are largely incomplete. 3- GI accession numbers for used sequences. Where no sequence was found or partial sequences were found, we indicate this by NF and P, respectively. 4 - Length of proteins used in this study. No length is given for partial sequences not used in the figures. 5 - A_Cb and A_Ce are sometimes refered to as W_Cb and W_Ce when refering to TRS130 sequences where these species have a large C-terminal deletion.

B: Additional genomes

Group[1]	Species	TRS120	TRS130	TRS65
Opisthokonta (FS)	*Candida guilliermondi3*[2]	+	+	+
Opisthokonta (FS)	*Candida lusitaniae*[2]	+	+	+
Opisthokonta (FS)	*Candida tropicalis*[2]	+	+	+
Opisthokonta (FS)	*Saccharomyces bayanus*[3]	+	+	+
Opisthokonta (FS)	*Saccharomyces kluyveri*[3]	+	+	+
Opisthokonta (FS)	*Saccharomyces kudriazevii*[3]	+	+	+
Opisthokonta (FS)	*Saccharomyces mikatae*[3]	+	+	+
Opisthokonta (FS)	*Saccharomyces paradoxus*[3]	+	+	+
Opisthokonta (FP)	*Aspergillus fumigatus*[4]	+	+	+
Opisthokonta (FP)	*Aspergillus oryzae*[5]	+	+	+
Opisthokonta (FP)	*Aspergillus terreus*[2]	+	+	+
Opisthokonta (FP)	*Podospora anserina*[6]	+	+	+
Opisthokonta (FB)	*Phanerochaete chrysosporium*[7]	+	+	**NF**
Opisthokonta (Fungi)	*Rhizopus oryzae*[2]	+	+	+
Opisthokonta (Fungi)	*Encephalitozoon cuniculi*[6]	**NF**	**NF**	**NF**
Opisthokonta (Fungi)	Sclerotinia sclerotiorum	+	+	+
Chromalveolata	*Phytophthora ramorum*[7]	+	+	**NF**
Chromalveolata	*Phytophthora sojae*[7]	+	+	**NF**
Chromalveolata	*Thalassiosira pseudonana*[7]	+	+	**NF**
Chromalveolata	*Plasmodium falciparum*[4]	+	+	+
Amoebazoa	*Entamoeba histolytica*[4]	**NF**	**NF**	**NF**
Excavata	*Trypanosoma cruzi*[4]	+	+	+
Archaeplastida	*Cyanidioschyzon merolae*[8]	**NF**	**NF**	**NF**

1- Phylogentic groups. FS, FP, and FB are abbreviations for these groups used in Figure 1 and Table S1A. Other group names reflect the super-groups listed in (ADL *et al.* 2005). The sources of the additional genomes are 2- http://www.broad.mit.edu.
3- http://www.yeastgenome.org/. 4- http://www.tigr.org.
5- http://www.bio.nite.go.jp/ngac/e/rib40-e.html.
6- http://www.genoscope.cns.fr/externe/English/Projets. 7- http://genome.jgi-psf.org.
8- http://merolae.biol.s.u-tokyo.ac.jp/ Sequences are indicated as being present with a '+' or absent by an 'NF'.

C: TRAPP I-specific subunits in genomes lacking Trs120 and Trs130

Group	*Species*	Bet3	Bet5	TRS20	TRS23	TRS31	TRS33
Archaeplastida	*Cyanidioschyzon merolae*[8]	+	+	**NF**	+	+	+
Amoebazoa	*Entamoeba histolytica*[4]	+	+	+	+	**NF**	+
Opisthokonta (Fungi)	*Encephalitozoon cuniculi*[6]	**NF**	**NF**	**NF**	+	+	**NF**

See part B for explanation.

Table S2: Trs120 highly conserved (HC) amino acids

Domain[1]	Hs[2]	Sc[2]	Hits[3]	A Am	Gg	Xl	M Dd	P At	Os	FS Ag	Ca	Cg	Dh	Kl	Sc	Yl	FP Nc	FB Cn	Um	FZ Sp	AI Tt
I	169	87	30F+I+4L															K			
	170	88	16Q+N+5D+14E																		
	173	91	2K+34R																		
	178	96	6I+13L+15V															-	A		
	179	97	21I+7L+6V															-			T
	181	99	15I+8L+10V	F														-			Q
II	302	274	34R															P			L
	312	284	F+34L																		Q
	314	286	3S+28A+3T															D			L
	315	287	34G															S			M
	320	292	8S+27A																		K
*	336	308	34D+2E																		
*	339	311	36W																		
	343	315	3S+29A+2T															N			Q
*	345	317	4D+30E															S			A
	353	325	8M+11I+13L+2V													Q					C
III	477	505	9M+3I+21L				-											-			D
	496	524	F+M+15I+17L													V		G			
	500	528	K+34R															T			
	501	529	R+32K															D		L	S
	504	532	28F+4M+I															D	H	K	
	507	535	Q+33R													Y		T			
IV	692	724	M+3I+3L+26V				A					-									P
	705	737	Q+D+34N																		
*	710	742	Q+9D+23E															S		S	K
	711	743	6I+19L+8V												F			E			T
	713	745	16I+3L+17V																		
	716	748	11M+12I+9L+3V															R			
	718	750	F+2I+32L																		E
	746	773	7I+14L+12V								K		A					S			
	755	781	M+8I+16L+9V											F							E
	824	865	M+5I+23L+6V																	F	
	827	868	33G															A		Y	Q
	834	875	21I+L+11V							C										Y	N
	836	877	M+6I+22L+4V				F	F													S
*	838	879	D+34N															G			
V	1004	1040	2M+3I+27L+V					R	R												A
	1006	1042	3I+27L+3V			F	F											S			
*	1007	1043	2N+30D+2E															G	H		
*	1010	1046	33N					L	L									G			
	1028	1068	2M+13I+16L+4V															E			
	1035	1075	K+32R				T											V		N	
	1038	1078	13I+9L+11V														F	S			Y
	1080	1131	4I+20L+9V		Q													-			F
*	1084	1135	33W+Y		E													-			
V+	1183	1235	34G															-			D

1- Domains shown in Figure 2. V+ indicates a highly conserved amino acid beyond domain Trs120-V. Asterisks in this column highlight very dramatic amino acid conservation; 2- Position of the highly conserved amino acids in *H sapiens* (Hs) or *S. cerivisiae* (Sc) coordinates; 3- Similar amino acids at the alignment position. Conserved amino acid groups are defined in the main text; Subsequent columns show any exceptions to the conservation shown in the ‘Hits’ column.

Table S3: Trs130 highly conserved (HC) amino acids

					A		W		M	P		FS							FP	FB		FZ
Domain[1]		Hs[2]	Sc[2]	Hits[3]	Dm	Dp	Cb	Ce	Dd	At	Os	Ag	Ca	Cg	Dh	Kl	Sc	Yl	Ci	Cn	Um	Sp
I		29	20	2I 17L 9V			S	S												-		
		30	21	20F 4W 5Y																-	-	
		37	28	5F 8I 18L																		
		41	32	3F 4I 21L						Q			-						-			
	*	48	39	28W						L	L		-									
		65	56	20F I 8L	A	T																
		89	76	3M 18I 4L 4V												-	-					
	*	98	85	16D 14E								-										
	*	100	87	2F 29Y																		
		101	88	10R 21K																		
		105	92	22R 9K																		
		108	95	M 15I 10L 4V																		-
	*	111	98	29W						I	I											
		126	118	M 6I 8L 16V																		
		146	138	8I 5L 16V									-									-
		148	140	15D 13E						A	A									-		
		149	141	3R 28K																		
		150	142	14I 11L 3V												-	-				-	
	*	153	145	31D																		
	*	154	146	31F																		
		165	157	13L 15V					R		F							-				
		182	171	11F M 17L				V				-										
		186	175	5M 11I 14L V																		
		187	176	2Q 16K 10R						T	V											-
		190	179	4M 10I 12L 3V																-	-	
	*	202	191	2Q N 2D 23E									-			-						-
		205	194	5M 19I 6L V																		
		223	207	26F 3L									-			-						
		226	210	H 10Q 2R 15K			M	M					-									
		229	213	28L			Y	Y								-						
	*	241	225	22D 7E			Y	Y														
		242	226	5S 25A									-									
		247	231	Q 2N 22D 3E					F					-		-						
		249	233	2M 3I 26L																		
		253	237	12F M I 14L						Y	Y							-				
		296	267	22I 5L 2V								-	-									
		304	275	19F 11L								-										
		306	277	17F 2M 11L												-						
		307	278	H Q 3K 23R								-		-								-
	*	309	280	2F 26Y										-	-	-						
		310	281	10I 17L 2V										-			-					
	*	311	282	28F 3L																		
		314	285	26Q K 2E													-					-
		317	288	3M 26L									-					-				
II		444	392	F 3M 25L										-				-				
		447	395	4S 21A 4T											-			-				
		448	396	3F 5I 22L										-								
	*	454	402	28F Y			V	V														
	*	458	406	6F 22Y			N	N								-						

1- Domains shown in Figure 3; Other notes as in Table S2

Table S4: Trs65 highly conserved (HC) amino acids

				FS					FP					FB
Domain[1]		Sc[2]	Hits[3]	Ag	Ca	Kl	Sc	Yl	Af	An	Cg	Ci	Gz	Cn
I		1	F 3M 2I 8L					S		-				
		5	5I 4L 6V							-				
		26	15R							-				
II		96	8I 3L 4V						-					
	*	97	4F 11W						-					
		103	4M 6L 5V						-					
III	*	286	16P											
		287	F 6I 8L							V				
	*	293	15D E											
		297	5F M 3I 6L				A							
		299	2F 14Y											
		301	F I 13L											-
		315	9I 6L V											
		317	9I 5L V											-
		331	M 7I 3L 4V											-
	*	335	16W											
		339	5I 5L 6V											
		341	11F 4L	S										
IV		424	15G									A		
		431	2I 5L 9V											
	*	435	15N											-
		437	13S 2T										-	
		441	4F M 2I 9L											
		443	3M I 11L V											
		500	6I 6L 3V								A			
		501	7I 3L 5V								S			
		503	I 14L								P			
	*	506	N 13D E								R			
		508	9R 6K								S			
		512	M 2I 12L V											
	*	514	15P								-			
		522	3I 12L								-			
		522	3I 12L								-			
		524	7F 2M I 4L	C							-			
		525	6I 7L 2V								-			
		527	F 4I 9L		V						-			
		530	15G								-			
		535	4I 10L			T					-			
		535	4I 10L			T					-			
		538	M 2I 4L 7V							K	-			
		539	6R 8K							C	-			
		540	5I 3L 6V							D	-			
		557	M 7I L 5V							N	-			

1- Domains shown in Figure 3; Other notes as in Table S2

Supplementary References

ADL, S. M., A. G. SIMPSON, M. A. FARMER, R. A. ANDERSEN, O. R. ANDERSON *et al.*, 2005 The new higher level classification of eukaryotes with emphasis on the taxonomy of protists. J Eukaryot Microbiol **52:** 399-451.

ALTSCHUL, S. F., T. L. MADDEN, A. A. SCHAFFER, J. ZHANG, Z. ZHANG *et al.*, 1997 Gapped BLAST and PSI-BLAST: a new generation of protein database search programs. Nucleic Acids Res **25:** 3389-3402.

COX, R., R. J. MASON-GAMER, C. L. JACKSON and N. SEGEV, 2004 Phylogenetic analysis of Sec7-domain-containing Arf nucleotide exchangers. Mol Biol Cell **15:** 1487-1505.

SODING, J., 2005 Protein homology detection by HMM-HMM comparison. Bioinformatics **21:** 951-960.
